# Genome Sequence of an Early Imported Case of SARS-CoV-2 Delta Variant (B.1.617.2 AY.122) in Iraq in April 2021

**DOI:** 10.1128/mra.00977-22

**Published:** 2022-10-17

**Authors:** Nihad A. M. Al-Rashedi, Hussein Alburkat, Murad G. Munahi, Ali H. Jasim, Batool Kadham Salman, Basel Saber Oda, Abbas Abd-Alsahib Mossa, Ahmed Abdulrazzaq Abbas, Olli Vapalahti, Tarja Sironen, Teemu Smura

**Affiliations:** a Department of Biology, College of Science, Al-Muthanna University, Samawah, Iraq; b Department of Virology, Faculty of Medicine, University of Helsinki, Helsinki, Finland; c Department of Public Health, Al-Muthanna Health Directorate, Samawah, Iraq; d Directorate of Medical Affairs, Ministry of Health, Baghdad, Iraq; e Department of Public Health, Thi-Qar Health Directorate, Nasiriyah, Iraq; DOE Joint Genome Institute

## Abstract

The severe acute respiratory syndrome coronavirus 2 (SARS-CoV-2) Delta variant was first reported in India. Thereafter, the Delta variant became the most prevalent variant globally. Here, we report the complete genome sequence of an early imported case of a SARS-CoV-2 B.1.617.2 AY.122 strain in Iraq. The strain was obtained from a flight passenger from India to Iraq on 20 April 2021.

## ANNOUNCEMENT

Severe acute respiratory syndrome coronavirus 2 (SARS-CoV-2) belongs to the genus *Betacoronavirus*, family *Coronaviridae*, and emerged in Wuhan, China, causing the coronavirus disease 2019 (COVID-19) epidemic throughout the world. Since the COVID-19 outbreak began in December 2019, several SARS-CoV-2 variants have emerged and circulated globally (1). Through sequencing-based surveillance and epidemiological studies, genetic lineages of SARS-CoV-2 have been classified into variants of concern (VOCs) and variants of interest (VOIs) (2).

We analyzed a coding-complete genome sequence of a SARS-CoV-2 Delta variant imported to Iraq from India in April 2021. A 72-year-old man who lived in Thi-Qar province, Iraq, traveled to India and then returned to Iraq on 20 April 2021. The first symptoms of fever, headache, and muscle pain manifested on 22 April 2021. A nasopharyngeal swab sample was added to AccuZol reagent (Bioneer Pacific, Taejeon, South Korea) at a 1:3 (vol/vol) ratio. Viral RNA was extracted following the manufacturer’s protocol. On 23 April 2021, the sample tested positive for SARS-CoV-2 using the STAT-NAT COVID-19 MULTI real-time PCR kit (Sentinel Diagnostics, Milan, Italy). The cDNA was synthesized using the LunaScript reverse transcription (RT) SuperMix kit (New England Biolabs, Ipswich, MA, USA) with random hexamers and oligo(dT), followed by SARS-CoV-2-specific PCR using the Q5 Hot Start high-fidelity 2× master mix (New England Biolabs) and xGen ARTIC v3 nCoV-2019 primers (Integrated DNA Technologies, Inc., Coralville, IA, USA). The PCR amplicons were purified using the Optima dye terminator removal (DTR) 96-well cleanup system (Edge BioSystems, San Jose, CA, USA), followed by sequencing library preparation using the NEBNext Ultra II library preparation kit for Illumina sequencing (New England BioLabs). The sample was sequenced using an Illumina NovaSeq 6000 system with a read length of 250 bp, which resulted in 1,222,270 reads. The sequence reads were trimmed and quality filtered using fastp (3) and assembled to the reference genome with BWA-MEM (4) implemented in the HaVoC pipeline (5). The CoVsurver (6) and Coronapp online servers were used to analyze the mutations in the SARS-CoV-2 genome, compared to the Wuhan-Hu-1/2019 sequence (GenBank accession number MN908947) (7). Lineages and clades were assigned using Pangolin v3.1.7 and the Nextclade webserver (Table 1) (8, 9). All software and tools were used with default parameters.

**TABLE 1:** Genomic features of the first recognized Delta variant case in Iraq^*a*^

Parameter	Finding
Virus name	hCoV-19/Iraq/Thi-Qar-1/2021
Sequence length (bp)	29,853
Mean coverage (×)	93,716
GC content (%)	38
Amino acid changes^*b*^	K81N, A488S, P1228L, P1469S, V167L, T492I, T77A, P323L, G671S, P77L, A394V, T19R, E156G, F157del, R158del, L452R, T478K, D616G, P681R, D950N, S26L, I82T, V82A, T120I, T40I, D119del, F120del, R203M, G215C, D377Y
GISAID clade	GK
Nextstrain clade	21J

a According to the coding-complete genome sequence of SARS-CoV-2, the patient was infected with the Delta variant (B.1.617.2 AY.122).

b The amino acid changes in the SARS-CoV-2 genome sequence, compared to the Wuhan-Hu-1/2019 sequence (GenBank accession number MN908947), were analyzed by using CoVsurver.

This study was approved by the Scientific Ethics Committees of the Al Muthanna University (approval number 8928 [30 July 2020]) and the Ministry of Health of Iraq (approval number D.A.F./1/1/4/121 [24 May 2021]).

### Data availability.

The genome sequence of this variant was deposited in the Sequence Read Archive (SRA) under the accession number SRX11518488, in GenBank under the accession number MZ555945, and in the GISAID database under the accession number EPI_ISL_2931131.
